# *In silico* Design of an Epitope-Based Vaccine Ensemble for Chagas Disease

**DOI:** 10.3389/fimmu.2019.02698

**Published:** 2019-11-22

**Authors:** Lucas Michel-Todó, Pedro Antonio Reche, Pascal Bigey, Maria-Jesus Pinazo, Joaquim Gascón, Julio Alonso-Padilla

**Affiliations:** ^1^Barcelona Institute for Global Health (ISGlobal), Hospital Clínic, University of Barcelona, Barcelona, Spain; ^2^Laboratory of Immunomedicine, Faculty of Medicine, University Complutense of Madrid, Madrid, Spain; ^3^Université de Paris, UTCBS, CNRS, INSERM, Paris, France; ^4^PSL University, ChimieParisTech, Paris, France

**Keywords:** Chagas disease, *Trypanosoma cruzi*, epitope-based, vaccine, CD4 T cell, CD8 T cell, B cell epitopes

## Abstract

*Trypanosoma cruzi* infection causes Chagas disease, which affects 7 million people worldwide. Two drugs are available to treat it: benznidazole and nifurtimox. Although both are efficacious against the acute stage of the disease, this is usually asymptomatic and goes undiagnosed and untreated. Diagnosis is achieved at the chronic stage, when life-threatening heart and/or gut tissue disruptions occur in ~30% of those chronically infected. By then, the drugs' efficacy is reduced, but not their associated high toxicity. Given current deficiencies in diagnosis and treatment, a vaccine to prevent infection and/or the development of symptoms would be a breakthrough in the management of the disease. Current vaccine candidates are mostly based on the delivery of single antigens or a few different antigens. Nevertheless, due to the high biological complexity of the parasite, targeting as many antigens as possible would be desirable. In this regard, an epitope-based vaccine design could be a well-suited approach. With this aim, we have gone through publicly available databases to identify *T. cruzi* epitopes from several antigens. By means of a computer-aided strategy, we have prioritized a set of epitopes based on sequence conservation criteria, projected population coverage of Latin American population, and biological features of their antigens of origin. Fruit of this analysis, we provide a selection of CD8^+^ T cell, CD4^+^ T cell, and B cell epitopes that have <70% identity to human or human microbiome protein sequences and represent the basis toward the development of an epitope-based vaccine against *T. cruzi*.

## Introduction

Chagas disease is a devastating neglected disease that affects several million people worldwide (1). It is caused by the protozoan parasite *Trypanosoma cruzi* (*T. cruzi*; order: Kinetoplastida; family Trypanosomatidae). The parasite is transmitted by triatomine insect vectors (order Hemiptera; family Reduviidae) endemic to the Americas, and through vector-independent routes such as blood transfusion or mother-to-child (1). The latter are of relevance in endemic and non-endemic regions alike where the disease has been globalized in recent decades due to population movements (2).

There are two drugs to treat the infection: benznidazole (BNZ) and nifurtimox (NFX). Although highly efficacious against the short (8–12 weeks long) acute stage of the disease, this stage is mostly asymptomatic and therefore it generally goes undiagnosed and untreated. It is at the chronic stage, which can last from years to decades, when the onset of symptoms occurs. It is usually then when the infection is diagnosed and treatment is provided, but unfortunately, the drugs' efficacy at this stage is reduced (1). Moreover, BNZ and NFX long dosage regimes have severe toxicity associated, which can lead to treatment discontinuation and failure (3, 4). Without treatment or upon its failure, about 30% of the chronically infected people will suffer incapacitating heart and/or gut tissue disruptions that can be life-threatening (1). If the damage to the tissues is advanced, diagnosis and treatment might arrive too late to reverse the situation (5). In view of present deficiencies to get access to diagnosis and treatment, a Chagas disease vaccine that could either prevent the infection or the development of the symptomatology would mean a major breakthrough in the management of the disease.

Two parasitic forms co-exist in *T. cruzi*-infected humans: amastigotes replicate intracellularly in multiple host cell types and transform into circulating motile trypomastigotes that spread the infection upon bursting the host cells. The existence of these two forms in the intracellular and extracellular domains requires a broad, complete, active, and robust parasite-specific immune response to control the infection. Despite the immune system's intervention, some parasites manage to escape and, at a simple conceptual level, this is how the parasite persists (6). Recently, parasite dormancy has also been described as another mechanism of immune evasion (7). The aim of a vaccine for Chagas disease will be to induce a specific and protective immune memory against the parasite able to control the infection and halt the tissue damage driven by the parasite.

Experimental animal models and studies with clinical samples have shown that cellular immunity mediated by antigens specific for CD8^+^ T cells are essential to control the infection (8). Activated CD4^+^ T cells are critical for the secondary expansion of CD8^+^ cytotoxic T cells and their efficient cytotoxic activity. Moreover, the relevance of CD4^+^ T cell-mediated immunity during *T. cruzi* infection was demonstrated when deletion of this cell population in infected mice resulted in increased parasitemia and exacerbation of pathology (9), as well as considering that in Chagas disease patients co-infected with HIV, a decrease in the amount of CD4^+^ T cells correlates with increased parasitic loads (10). Besides, parasite-specific B cell responses have been reported as fundamental to keep a systemic anti-parasitic response and prevent exhaustion of parasite-specific CD8^+^ T cells (11), plus parasite-specific antibodies are crucial to halt the infection of new cells by free-swimming trypomastigotes (12).

Several vaccine candidates against *T. cruzi* infection have been tested in the last decades (13). The majority of them were based on the delivery of single antigens or fragments of single antigens either as recombinant protein subunits or vectored by recombinant viruses or bacteria (13). In addition, other approaches have relied on chimeric protein constructions as immunogens (14, 15). The most advanced candidates have reached evaluation in dog (16, 17) and non-human primate (18) models of *T. cruzi* infection or have been shown to *ex vivo* recall memory responses in chronic Chagasic patients from Mexico (19), but none has already gone beyond preclinical developmental stages.

Two parasite-related issues greatly hamper vaccine development for Chagas disease: the wide phenotypic diversity of the parasite, and its high biological complexity. *T. cruzi* strains infecting humans are classified in six different genotypes or Discrete Typing Units (20) and can commonly cause mixed infections (21, 22). Moreover, each parasite strain has more than 10,000 protein coding genes (CDS) per haploid genome (23). Thus, the first step of the vaccine development process, i.e., the selection of the desired antigens that will be used as immunogens, is a hard-to-navigate crossroads. Aimed at increasing the chances of progression into clinical testing, we have devised a strategy that might enable overcoming this inconvenience. It is based on an epitope-based vaccine design that would allow the delivery and evaluation of multiple *T. cruzi* epitopes from multiple parasitic antigens in one single construct. Building on an emerging paradigm of rational epitope-based vaccine design (24), we have applied tailor-made and publicly available immunoinformatics resources to make a selection of parasite-specific CD8^+^ T cell, CD4^+^ T cell, and B cell epitopes, prioritized accordingly to sequence conservation criteria, projected protection coverage (PPC) in populations of Latin America, and lack of identity to human genome and human microbiome proteins. Similarly to other studies (25–28), all the epitopes selected were either validated or predicted in the human immunological context. Such strategy could be useful toward the design and further development of vaccine candidates against complex pathogens. In this case, in an attempt to anticipate an epitope-based *T. cruzi* vaccine ensemble, we provide a set of 18 CD8^+^ T cell epitopes with >85% PPC in Latin America, 2 CD4^+^ T cell epitopes with >99% coverage in Latin American populations, and 10 B cell epitopes from exposed antigens.

## Materials and Methods

### Collection of *T. cruzi*-Specific Epitopes

T and B cell epitope sequences were downloaded from the Immune Epitope DataBase and Analysis Resource (IEDB; URL: http://www.iedb.org/) (29). We retrieved *T. cruzi* (“antigen ID 5693”) epitopes from positive assays, which had been elicited in the course of Chagas disease (“infectious disease”) in humans. Major histocompatibility complex (MHC) Class I (CD8^+^ T cell epitopes), MHC Class II (CD4^+^ T cell epitopes), and B cell epitopes were collected separately. Information on their antigens of origin was obtained too. We only considered CD8^+^ T cell epitopes with nine residues as the majority of known epitopes processed by MHC I are 9-mers (30). Any non-peptidic and/or duplicate sequences were discarded from further analysis.

### Collection of *T. cruzi* Protein Sequences

We downloaded all available *T. cruzi* protein sequences in their Open Reading Frame format (ORF_AA) from the eight parasite strains in TriTrypDB resource release 35 (31). These are (DTU provided in parenthesis): CLBrener Non-Esmeraldo-like (TcVI), CLBrener Esmeraldo-like (TcII), CLBrener (TcVI), Dm28c (TcI), Esmeraldo (TcII), JRcl4 (TcI), SylvioX10 (TcI), and Tulacl2 (TcVI). A single file containing all the sequences (~1 × 10^7^ ORF_AA FASTA entries) was generated. We excluded the bat isolated Marinkellei strain due to its phylogenetic distance to the others (32). Rationale for the data download as ORF_AA is that we wanted to include all *T. cruzi* strains available in TriTrypDB release 35, including JRcl4 and Tulacl2 strains, which lack annotation details.

### Generation of Clusters and Multiple Sequence Alignments of *T. cruzi* Protein Sequences

We used CD-HIT, running stand-alone with default settings, to reduce the redundancy of *T. cruzi* proteins clustering them with a shared identity >90% (33). The resulting ~1.36 × 10^6^ clusters were filtered down by means of custom scripts to retain only those that contained protein sequences from at least seven different strains. This way, we ensured that clusters considered for further analysis represented the protein diversity among almost all strains available. Protein sequences within each of the remaining 13,571 clusters were aligned using MUSCLE (software version 3.8.31) (34) to obtain an equivalent number of multiple sequence alignments (MSA) with their corresponding consensus sequences. Instead of the consensus sequence, built with pieces from different sequences and thus unreal, we kept as reference of each MSA the sequence in the alignment that was most similar to the consensus one.

### Generation of *T. cruzi* Invariable Proteome and Identification of Conserved IEDB Epitopes

The Shannon entropy (*H*) parameter (35) was used to measure sequence variability on every position of the MSA according to the equation (Equation 1):

(1) $$H=-\sum_{i}^{M}P_{i}\;{\text{log}}_{2}(P_{i}),$$

where *P*_*i*_is the frequency of an amino acid of type *i*, and *M* is the number of total different amino acids (20). *H* = 0 is equivalent to no variation in a given position among the studied protein sequences within the alignment, whereas higher values correspond to higher variation in that position (36). Gaps were considered as data. Those residues at positions with an entropy value of *H* > 0.5 or *H* > 1.0 were masked in the assigned protein reference sequence, substituting the residue symbol by an asterisk symbol (^*^). As a result, we obtained two masked FASTA files (one for each entropy threshold) that represented the conserved proteome of *T. cruzi* at those two levels of entropy.

### Prediction of T Cell Epitopes

For *de novo* prediction of CD8^+^ T cell epitopes (peptides), we used IEDB MHC I binding prediction algorithms (http://tools.iedb.org/mhci) on the *H* > 0.5 *T. cruzi*-masked proteome. Namely, these were ANN, NetMHCpan, NetMHCstabpan (all based on artificial neural networks) (37–39), CombLib_Sidney2008 (based on combinatorial peptide libraries) (40), PickPocket (based on the analysis of the MHC variable pocket residues) (41), SMM (stabilized matrix method) (42), and SMMPMBEC (a Bayesian extension of the former) (43). All methods were downloaded from IEDB and ran as stand-alone programs. We considered for further analysis just the 0.1% top scoring predicted epitopes from each tool that had been predicted by five or more different methods and submitted them to IEDB T cell Class I Immunogenicity predictor (http://tools.iedb.org/immunogenicity/) (44).

For the prediction of CD4^+^ T cell epitopes (peptides), we used the IEDB recommended method at MHC II binding predictions tool (http://tools.iedb.org/mhcii/) (45, 46). CD4^+^ T cell epitopes were predicted on the *T. cruzi*-masked proteome file (*H* > 0.5). We considered for further analysis only the 0.01% top scoring epitopes that were predicted to bind more than two human leukocyte antigen (HLA) alleles.

### Prediction of B Cell Epitopes

We followed two procedures for the prediction of B cell linear epitopes: (1) a structure-based prediction on surface antigens that contained at least one experimentally validated B cell epitope from IEDB, and (2) the B cell epitopes prediction tool BepiPred2.0 (47).

For (1), we elaborated a list with all the protein sequences that contained an experimentally validated epitope from IEDB and had been predicted to be surface exposed in the parasite. The list was blasted to the Protein Data Bank (PDB) database with BLASTP. Only one protein, the kinetoplastid membrane protein-11 (KMP11), had a positive hit that covered >80% of the queried sequence length. This was a KMP11 ortholog from the closely related *Trypanosoma brucei brucei* (PDB accession 5Y70) (48). Taking it as reference, we modeled the *T. cruzi* KMP11 onto it using MODELER (49). Then, we used the KMP11 model to perform relative solvent accessibility (RSA) calculations with NACCESS (50). Residues with RSA > 50% were considered as good candidates for being part of a potential epitope. We used PyMOL Molecular Graphics System, Version 1.8 Schrödinger, to visualize this B cell epitope on its modeled 3D structure.

For (2), we obtained at TriTrypDB a list of *T. cruzi* Esmeraldo-like proteins predicted to be surface exposed, either because they had a secretion signal peptide, or/and at least one trans-membrane domain, or/and a glycosylphosphatidylinositol (GPI) anchor signal. We manually curated the list taking into account annotation details to retain those proteins that were highly likely exposed, and we tagged them as “-exposed.” We fused them to the file containing all *T. cruzi* ORF_AA sequences to generate a file that included the “-exposed” tagged sequences as well. Again, we clustered all these sequences using CD-HIT with a shared identity threshold >90%, but this time, we kept for further analysis only those clusters with at least eight different strains that contained a “-exposed” tagged sequence. We then followed the steps outlined in sections Generation of Clusters and Multiple Sequence Alignments of *T. cruzi* Protein Sequences and Generation of *T. cruzi* Invariable Proteome and Identification of Conserved IEDB Epitopes to generate a masked (*H* > 0.5) *T. cruzi*-invariant “exposed” file. We identified the invariant regions of at least 15 residues long in it and created a FASTA file with the sequences of their corresponding antigens of origin. BepiPred2.0 was run on this file with a threshold set at >0.6 (47). We finally crossed the results of this prediction with the conservation results obtained from the previous procedure. Only those regions of at least 15 residues long that were predicted as epitopes by Bepipred2.0 and conserved accordingly to our analysis were considered as putative epitopes for subsequent prioritization.

### Prediction of T Cell Epitope HLA Binding Profiles and Computation of Their Projected Protection Coverage (PPC)

IEDB MHC I (38, 40, 42, 51) and MHC II (45, 46) binding predictors were used to calculate the binding profiles of CD8^+^ T cell and CD4^+^ T cell epitopes, respectively. We used the IEDB-recommended set of tools in both cases and a specific set of HLA I alleles with more alleles in it so as to better represent Latin American haplotypes (52) (Supplementary File 1) and the HLA II reference set provided by IEDB, respectively. In agreement with IEDB instructions, for MHC I epitopes, we kept for further analysis those predicted epitopes within a top percentile rank ≤ 1%. With respect to MHC II epitopes, we kept only those within the top 3% percentile rank.

We calculated the epitopes PPC with IEDB Population Coverage tool (http://tools.iedb.org/population/). PPCs of CD8^+^ T cell and CD4^+^ T cell epitopes for populations in Latin America was queried by “area—country—ethnicity,” selecting Mexico and South America (http://tools.iedb.org/population/). Throughout the paper, PPC is expressed as an average of the PPC computed for those two regions. We did not include Central America region in the calculation because it provided scarce information from very specific populations.

### Antigens Annotation, Blast Searches, and Other Analysis Procedures

We obtained the annotation data and biological features from the antigens of origin of the conserved putative epitopes at NCBI, UniProtKB, and TriTrypDB (31) in a sequential manner. First, we blasted the antigens' ORF_AA sequences (BLASTP at NCBI) to get their NCBI GeneInfo (GI) identifier and Accession numbers. We used the latter to query UniProtKB and retrieve the available information, including antigens' genes names at the best annotated CLBrener Esmeraldo-like strain. With those, we checked TriTrypDB Gene Page for further information retrieval (e.g., transcriptomics and proteomics experimental data).

In order to find out the epitopes' identity (% Id over queried sequence length) to proteins in humans, we blasted them against human microbiome protein sequences obtained from the NIH Human Microbiome Project (53) and against the NCBI non redundant (nr-) collection of human proteins. BLASTP was used with default parameters except for PAM30 Scoring Matrix and an expectation value (E-value) of 10,000.

We used SignalP, TargetP, and TMHMM (54) to predict patterns compatible with surface-exposed molecules on proteins with B cell epitopes. The possible presence of GPI-anchor signals in these was predicted at PredGPI website (55).

Proteasome processing of the proposed ensemble was predicted with NetChop using C-term 3.0 method and a threshold set at 0.7 (56). We used VaxiJen v2.0 to analyze the ensemble antigenicity (threshold at 0.5) (57), whereas its 3D structure was modeled with RaptorX web portal (58). Raptor X output also includes solvent accessibility (Acc) classification of residues as buried (B), medium (M), or exposed (E) (58). PyMOL Molecular Graphics System, Version 1.8 Schrödinger, was used to visualize the ensemble 3D structure.

## Results

### Identification of *T. cruzi* Conserved Experimentally Validated Epitopes at IEDB

We obtained 125 unique T cell epitopes (114 CD8^+^ T cell and 11 CD4^+^ T cell epitopes) and 2000 unique B cell epitopes. They are respectively listed in Tables S1A–C. The three lists of experimentally verified epitopes were crossed with the masked *T. cruzi* invariant proteome file with a threshold *H* > 1. Only the epitopes that perfectly matched invariant regions were considered hits. This way, we found three conserved CD8^+^ T cell epitopes (Table 1) and 104 conserved B cell epitopes (see Table S2). None of the 11 *T. cruzi* CD4^+^ T cell epitopes at IEDB was found to be conserved.

**Table 1:** Conserved *T. cruzi*-specific CD8^+^ T cell epitopes from IEDB.

**Epitope**	**Antigen** ** name^a^**	**Antigen ID^a^**	**Predicted HLA I profile^b^**	**PPC%^c^**	**Human hit (Id %)^d^**	**Human microbiome hit (Id %)^d^**
GVSGVINAL	Paraflagellar rod component	AAC32021.1	A^*^02:05, C^*^01:02, C^*^03:04	28.5	SJM29146.1 (77.8)	KWZ91528.1 (77.8)
VPEVTDVTL	Major paraflagellar rod protein	AAA30221.1	B^*^51:02, C^*^01:02	8.9	NP_001254479.2 (77.8)	EHM52480.1 (77.8)
KLEKIEDEL	Major paraflagellar rod protein	AAA30221.1	A^*^02:02, A^*^02:07	1.4	XP_016860742.1 (77.8)	EGN34163.1 (77.8)

a *Antigen name and ID number at NCBI database*.

b *Predicted HLA I binding profile obtained at IEDB (http://tools.iedb.org/mhci)*.

c *Average PPC for populations from Latin American origin obtained at http://tools.iedb.org/population/ as described in Materials and Methods*.

d *Blast hits in the NCBI human non-redundant protein collection and the Human Microbiome Project database are identified by their Accession reference number at NCBI; the percentage of identity (%Id, number of identical residues per queried length) of each epitope to its corresponding hit is shown in parenthesis*.

The three conserved CD8^+^ T cell epitopes were from two annotated antigens: major paraflagellar rod protein and paraflagellar rod component (Table 1). Their predicted binding profiles to HLA I were obtained and used to calculate their PPC. The epitope with the highest individual PPC was GVSGVINAL with 28.5% coverage; and the PPC of the combination of the three epitopes was 30.6%. This imposed the identification of more epitopes in order to achieve higher coverages. The need to search for other CD8^+^ T cell epitopes was further confirmed due to the >70% identity to human or human microbiome proteins of the three sequences (Table 1).

Regarding B cell epitopes, the majority of the hits were from non-exposed intracellular antigens (Table S2). However, B cell epitopes lead to the production of an antibody response, which is generally targeted against surface-exposed antigenic moieties. Thus, we restricted the selection of conserved B cell epitopes to those that originated from antigens predicted to be exposed, reducing the list to 5 epitopes from two antigens (Table 2). Four of the epitopes were from a lipophosphoglycan biosynthetic protein [within heat shock protein (HSP) 90 family], and the fifth was from the HSP70 *T. cruzi* protein. Since the large majority of IEDB *T. cruzi* B cell epitopes had been discovered by overlapping peptide arrays (59), the four epitopes at HSP90 antigen overlapped and were therefore fused into a single peptide sequence (Table 2). A transmembrane region spanning from positions 12 to 34 was predicted at the HSP90 antigen with TMHMM v2.0 (54), but the fused peptide within it locates to the outer exposed C-term of the molecule. No transmembrane regions were predicted in HSP70. Nonetheless, HSP70 epitope blasted with a very high identity to human genome and human microbiome proteins and we had to discard it from further consideration (Table 2). Thus, out of this “IEDB × INVARIANT PROTEOME” approach, we were just able to identify a single B cell epitope (GTDEGLLLPVDNDGDESS), which was unique and conserved, and had <70% identity to any protein sequence present in humans.

**Table 2:** Conserved *T. cruzi*-specific B cell epitopes at IEDB from antigens predicted to be exposed.

**Epitope^a^**	**Antigen name^b^**	**Antigen ID^b^**	**Antigen subcellular localization^c^**	**Human hit (Id %)^d^**	**Human microbiome hit (Id %)^d^**	**Evidence of exp.^e^**
GTDEGLLLPVDNDGDESS	Lipophosphoglycan biosynthetic protein/HSP90 superfamily	Q4DW89	S (2)	XP_024308866 (66.7%)	EHN62501.1 (44.4%)	Y/Y
ATNGDTHLGGEDFDN	Heat shock 70 kDa protein	P05456	S (1)	AAI12964.1 (93.0%)	EEX50322.1 (100%)	N/A

a *The underlined sequence is shared by four epitopes whose fusion yields this 18-amino-acid-long peptide*.

b *Antigen name and antigen ID were obtained from UniProtKB database*.

c *Subcellular localization of antigens was predicted with TargetP1.1 (54). S, secreted; the number in brackets stands for TargetP prediction “Reliability class” (RC), which ranges from 1 to 5, where 1 indicates the strongest prediction*.

d *Blast hits in the NCBI human non-redundant protein collection and Human Microbiome Project database are identified by their Accession reference number at NCBI; the percentage of identity (% Id, number of identical residues per queried length) of each epitope to its corresponding hit is shown in parenthesis*.

e *Evidence of expression in mammalian infective stages: existence (Y) or not (N) of evidence of expression by transcriptomics and/or proteomics as described in TriTrypDB search by Gene ID (31). N/A, not applicable since Hsp70 epitopes were not selected due to their high percentage of identity to protein sequences present in humans*.

### Prediction of T Cell Epitopes

T cells recognize epitopes when these are presented to them bound to MHC molecules. Therefore, epitopes can be predicted by computing their MHC-binding profile. Because of the differences in the molecular interactions between epitopes and MHC I and II complexes, the prediction of epitopes binding to MHC I is more accurate than to MHC II (60). For both types, we used IEDB tools as detailed in Materials and Methods.

We identified 127 predicted CD8^+^ T cell epitopes that were unique and originated from almost as many different *T. cruzi* conserved protein regions in the *T. cruzi H* > 0.5 masked proteome. In order to further cure this list, all sequences were submitted to the IEDB Class I Immunogenicity predictor (44), which rendered 89 epitopes with a immunogenicity scoring >0.0 (Table S3A). Subsequently, we blasted them against the non-redundant human protein collection and against the Human Microbiome Project collection of protein sequences. Only 18 out of those 89 peptides had identities <70% to any human or human microbiome hit and were considered for further analysis, which involved calculating their PPC (61) (Table 3). Individually, all of them had a PPC > 10%, whereas altogether, they provided a PPC of 88.3%. These 18 epitopes originated from as many as 17 different antigens with predominance of hypothetical proteins (13 out of 17; Table 3). The remaining four annotated antigens in the list were a phophoglycerate mutase, a putative DNA repair protein, a chaperonin HSP60, and a phosphatase-like protein (Table 3).

**Table 3:** Selection of predicted *T. cruzi*-specific CD8^+^ T cell epitopes.

**Epitope**	**Antigen name^a^**	**Antigen ID^a^**	**PPC (%)^b^**	**Human hit (Id %)**	**Microbiome hit^c^**	**Evidence of exp.^d^**
VYGRFYYRF	Phosphoglycerate mutase	XP_807293.1	52.5	NP_001316055.1 (55.6)	ETN46456.1	Y/Y
RFFPSVFWR	Hypothetical protein	XP_811816.1	46.2	EAW98638.1 (66.7)	EFR45113.1	Y/N
FPFCWLPTY	Hypothetical protein	XP_806973.1	39.1	NP_005305.1 (66.7)	ETN41230.1	Y/N
MPAFQGWAF	Hypothetical protein C3747_101g9	PWV07436.1	36.0	SJM35294.1 (66.7)	ERS97746.1	Y/N
KICHVVFFR	Hypothetical protein	XP_807600.1	34.7	NP_060595.3 (66.7)	EEG88082.1	Y^*^/Y
RTYHMIWNR	Hypothetical protein C3747_25g140	PWV16110.1	34.7	AIE56486.1 (55.6)	EGG52539.1	Y/N
RVFFWKVQR	Putative SNF2 DNA repair protein	PWV13412.1	34.7	AAN08628.1 (55.6)	EPH21217.1	Y/N
MTFVFEARR	Hypothetical protein	XP_817046.1	31.3	NP_653173.1 (66.7)	EFK35250.1	Y/N
KMWQRTFTR	Chaperonin HSP60, mitochondrial precursor	PWV11883.1	19.2	CCQ43203.1 (66.7)	EGJ45866.1	–
RLWRWRCMR	Hypothetical protein C3747_25g140	PWV16110.1	19.2	XP_011528872.1 (66.7)	EKY00389.1	Y/N
RINFCFYVR	Phosphatase-like protein	XP_809619.1	19.1	NP_001275914.1 (55.6)	EGF09027.1	Y/Y
RQRAILMYR	Hypothetical protein C3747_84g29	PWV08907.1	18.2	XP_005256351.1 (66.7)	EFV17694.1	Y/N
KMRVWRHQR	Hypothetical protein	XP_811814.1	15.3	XP_006723890.1 (66.7)	EGC78513.1	Y/N
RMNLITWHR	Hypothetical protein TCDM_03859	ESS67427.1	15.3	5WC2 (66.7)	EFC97415.1	Y/Y
FHDQTIFCL	Hypothetical protein	XP_805332.1	15.2	BAG10460.1 (55.6)	EUB31300.1	Y/N
MHDHYCFVL	Leucine carboxyl methyltransferase	XP_811822.1	15.2	NP_620154.2 (66.7)	KGF29870.1	Y^*^/Y
IPMRRRRSL	Hypothetical protein	XP_818330.1	15.0	NP_579877.1 (66.7)	EKU83050.1	Y/Y
FHFCITFCL	Hypothetical protein	XP_807567.1	11.3	EAW88842.1 (66.7)	EEG29914.1	Y/N

a *Antigen name and ID accession number at NCBI database*.

b *PPC for Latin America calculated at IEDB Population Coverage tool (61)*.

c *Percentage of identity of all microbiome hits blasted was 66.7%*.

d *Evidence of expression in mammalian infective stages: existence (Y) or not (N) of evidence of expression by transcriptomics and/or proteomics as described in TriTrypDB search by Gene ID (31)*;

* *the transcript expression value was higher in epimastigotes than in mammalian infective stages as described by Minning et al. (62)*.

For the prediction of CD4^+^ T cell epitopes, we provided the *T. cruzi*-masked (*H* > 0.5) proteome file to the IEDB MHC II Binding Predictor (45, 46). In total, 2,497 potential epitopes reported to bind from four to one distinct HLA II allele were retrieved. We kept for further analysis the 17 *T. cruzi*-specific CD4^+^ T cell peptides predicted to bind at least three distinct HLAs, which also had identities to proteins in humans below the 70% threshold (Table S3B). We then subjected them to PPC computation and ultimately selected two CD4^+^ T cell epitopes that individually provided 99.5 and 38.4% PPC, whose combination was shown to project 99.8% coverage (Table 4).

**Table 4:** Selection of predicted *T. cruzi*-specific CD4^+^ T cell epitopes.

**Epitope**	**Antigen name^a^**	**Antigen ID^a^**	**PPC (%)^b^**	**Human hit (Id %)^c^**	**Microbiome hit (Id %)^c^**	**Evidence of exp.^d^**
DDELFHYFLWTFFFIDLLYAVM	Hypothetical protein	XP_816368.1	95.9	BAC87213.1 (52.3)	ERH31153.1 (43.91)	Y/N
YIFIECFQIMRAFRLRGASFF	Co-chaperone GrpE	XP_804093.1	38.4	SJM30315.1 (28.6)	EJN84302.1 (47.6)	Y^*^/Y

a *Antigen name and ID number at NCBI*.

b *PPC for Latin America calculated at IEDB Population Coverage tool (61)*.

c *Percentage of identity is given in parenthesis and stands for the number of identical residues per queried length of each epitope to its corresponding blasted hit*.

d *Evidence of expression in mammalian infective stages: existence (Y) or not (N) of evidence of expression by transcriptomics and/or proteomics as described in TriTrypDB search by Gene ID (31)*;

* *the transcript expression value was higher in epimastigotes than in mammalian infective stages as described by Minning et al. (62)*.

### Prediction of B Cell Epitopes

**Figure 1:**
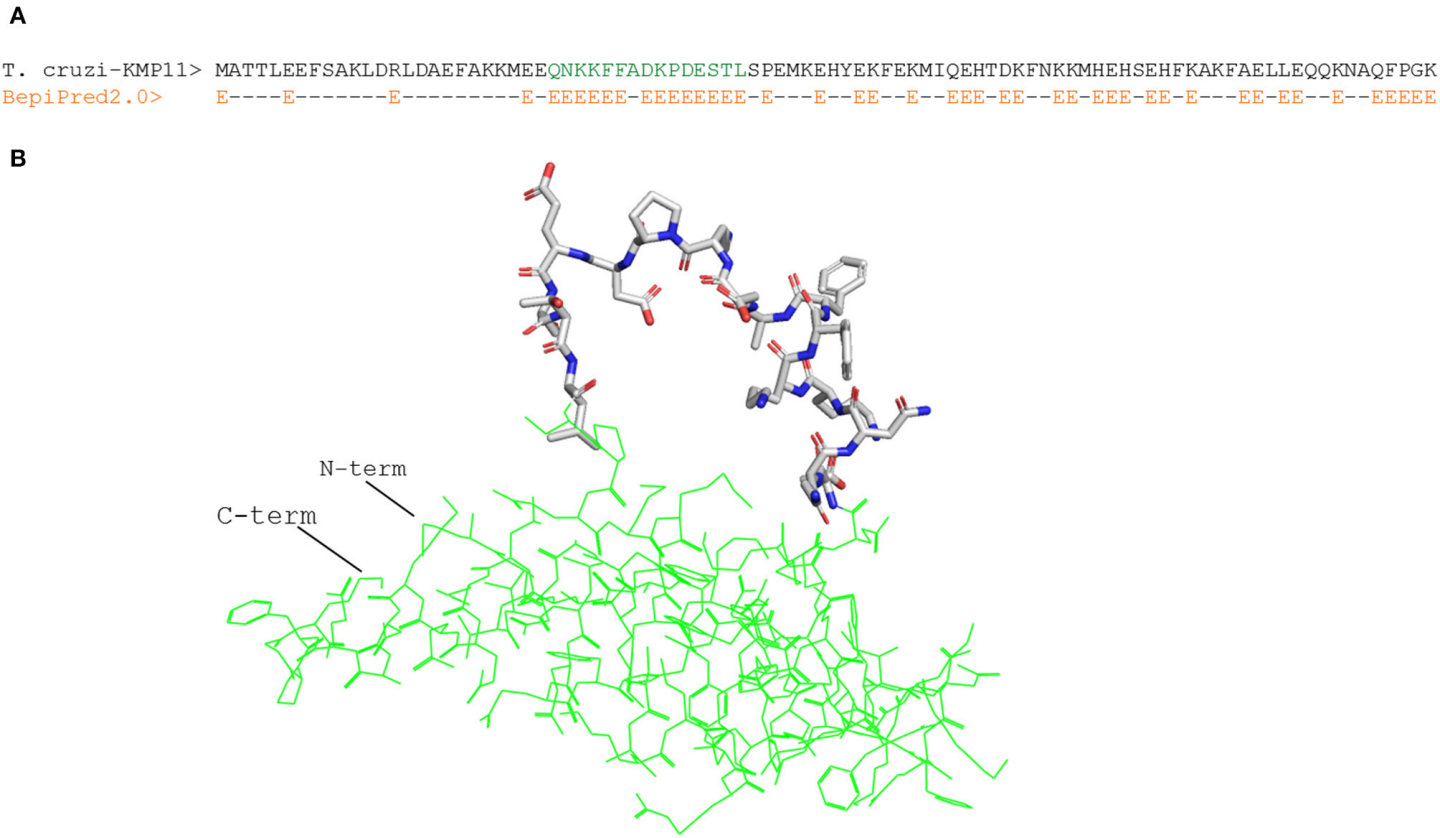
Structure-predicted B cell epitope in *T. cruzi* KMP11 antigen. **(A)** Alignment of *T. cruzi* KMP11 sequence with the BepiPred2.0 prediction of the likelihood of each residue of being part of an epitope (E). **(B)** PyMOL rendered picture of the *T. cruzi* KMP11 model with the predicted epitope QNKKFFADKPDESTL shown as sticks and the KMP11 background featured as pale yellow ribbon. The N-term and C-term extremes of the protein are indicated.

We focused on linear B cell epitopes because they can be delivered isolated from their antigen context to induce selective humoral responses. We applied two methods to predict B cell epitopes: a structure-based approach and a sequence-based approach with the tool Bepipred2.0 (47). With the former, we identified a potential B cell epitope in the parasite KMP11 protein (Figure 1A), an antigen that has been thoroughly researched (63, 64). We identified a region of 15 residues long with all but one fulfilling the RSA threshold (RSA > 50%). Visualization of this region in the *T. cruzi* KMP11 3D model illustrates its accessibility as the peptide clearly points away from the rest of the molecule (Figure 1B). The epitope was shown to have <70% identity to proteins in humans (Table 5), and an alignment of all the homologous sequences in the file containing all ORFs_AA used in the study indicated that it was highly conserved in seven out of the eight *T. cruzi* strains from TriTryDB release 35 used in the study (Supplementary File 2).

**Table 5:** Selection of predicted *T. cruzi*-specific B cell epitopes.

**Epitopes**	**Antigen name^a^**	**Antigen ID^a^**	**Antigen subcellular localization^b^**	**Human hit^c^ (Id %)**	**Human microbiome hit^c^ (Id %)**	**Evidence of exp.^d^**
QNKKFFADKPDESTL	Kinetoplastid membrane protein 11 (KMP11)	XP_810488.1	– (1)	AAH42613.1 (56.0%)	EOQ36117.1 (62.5%)	Y^*^/Y
EGESRHRTRSGSARHHRRHHRNEAGG	Hypothetical protein, conserved	XP_814578.1	−4	XP_006721247.1 (57.7)	EFX53962.1 (57.7)	Y/Y
RRRRHSRSKRGEEDGGET	Hypothetical protein, conserved	XP_814578.1	−4	XP_016883994.1 (55.6)	EEO54893.1 (61.1)	Y/Y
GTPSRTTGRSTSTTRGVSRPTNGVTPSTSLAHRASTPGRTGTRSTTPSRSSVLS	Associated kinase of Tb14-3-3, putative	XP_819464.1	S (1)	–	XP_016882982.1 (24.1)	Y/Y
GVCTSAEPRDLLDPVALCMPYPGAERIIG	Associated kinase of Tb14-3-3, putative	XP_819464.1	S (1)	AAI12922.1 (44.8)	EET82433.1 (37.9)	Y/Y
NSQETPDQQKTGITRV	Associated kinase of Tb14-3-3, putative	XP_819464.1	S (1)	AAY14773.1 (37.5)	KZA05920.1 (68.8)	Y/Y
REARGTSTPRRAETPSGGSRVRGA	Associated kinase of Tb14-3-3, putative	XP_819464.1	S (1)	NP_573568.1 (37.5)	OFQ81576.1 (54.2)	Y/Y
KRSRSHNDGPARKRRRKDNRP	Methyl-transferase, putative	XP_803963	S (5)	CAD38887.1 (61.9)	EFD49634.1 (47.6)	Y/N
NPSASPEASWQLNQSWNPL	Hypothetical protein, conserved	XP_809003.1	S (5)	EAW82987.1 (52.6)	EHS88047.1 (63.2)	Y/N

a *Antigen name and Accession reference obtained at NCBI database upon BLASTP analysis of the corresponding ORF_AA sequence as detailed in Materials and Methods*.

b *Antigen subcellular localization was predicted with TargetP1.1 (54); S, secreted; -, any other location; the number in parentheses stands for TargetP prediction “Reliability class” (RC), which ranges from 1 to 5, where 1 indicates the strongest prediction*.

c *Percentage of identity is given in parenthesis and stands for the number of identical residues per queried length of each epitope to its corresponding blasted hit*.

d *Evidence of expression in mammalian infective stages: existence (Y) or not (N) of evidence of expression by transcriptomics and/or proteomics as described in TriTrypDB search by Gene ID (31)*;

* *the transcript expression value was higher in epimastigotes than in mammalian infective stages as described by Minning et al. (62)*.

The Bepipred2.0 approach managed to identify other 10 potential B cell epitopes (Table S3C). They all had <70% identity to proteins present in humans. Nonetheless, a closer inspection showed that the antigens of two of them were predicted to localize at the mitochondrion and thus they were discarded from further analysis. The remaining eight peptides originated from four distinct antigens that were mostly predicted to be secreted (Table 5). Moreover, there was experimental evidence at the transcriptomics level indicating that they were preferentially expressed at the parasite mammalian infective stages (Table S3C).

### Proposed Epitope-Based Vaccine Ensemble

**Figure 2:**
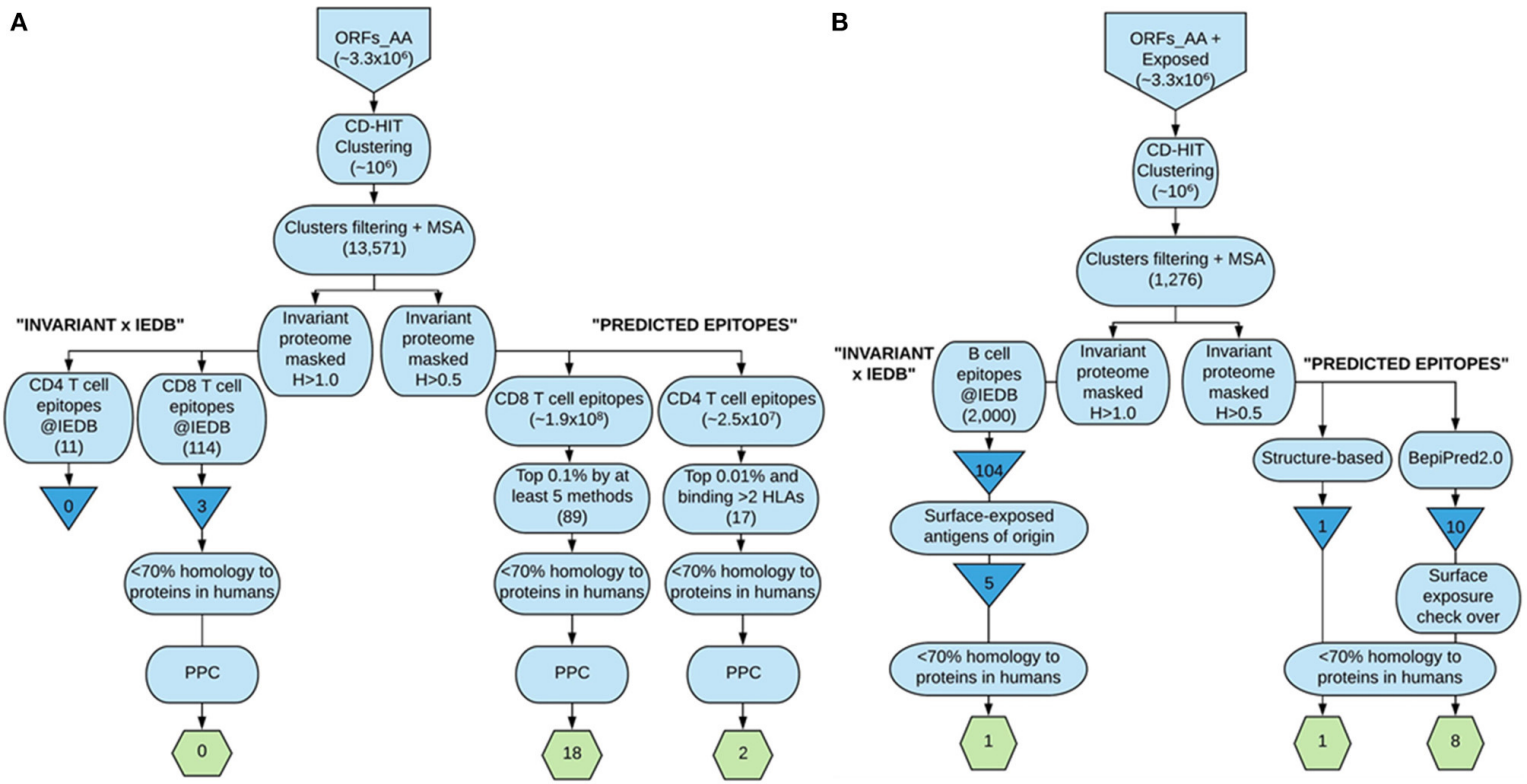
Flowchart summary of the epitope identification and prioritization steps followed in our strategy. **(A)** depicts the steps followed to arrive at the selected CD8^+^ and CD4^+^ T cell epitopes included in the ensemble proposed, whereas **(B)** shows the strategies undertaken to select the B cell epitopes finally included. The larger boxes show the steps; triangles show IEDB-derived *T. cruzi*-specific unique epitopes, and the hexagons contain the finally selected epitopes. This figure was made at https://www.lucidchart.com.

We ultimately provide the selection of epitopes that could ensemble a potential epitope-based vaccine against *T. cruzi* that fulfill the criteria of sequence conservation, widespread PPC, and lack of identity to proteins present in humans (Table 6). The list includes 18 CD8^+^ T cell epitopes from 17 distinct antigens, 2 CD4^+^ T cell epitopes from 2 antigens, and 10 B cell epitopes from 6 antigens. In total, there are 30 epitopes from 25 antigens of the parasite. All of them but one B cell epitope were predicted (Table 6). The steps followed to arrive at these 30 epitopes are depicted in Figure 2, which summarizes the work done and the selection procedures performed toward the prioritization of the CD8^+^ and CD4^+^ T cell epitopes (Figure 2A), as well as the B cell epitopes (Figure 2B) finally included in the ensemble. Although we have used *T. cruzi* as our target organism, the strategy followed could be employed to identify epitopes of interest toward the design and further development of vaccine candidates against other complex pathogens, for instance, *Leishmania* spp.

**Table 6:** Proposed epitope vaccine ensemble for *T. cruzi*.

**Epitopes**	**Antigen name^a^**	**Antigen ID^a^**	**Src^b^**
**CD8+** **T CELL EPITOPES VACCINE COMPONENT**			
VYGRFYYRF, RFFPSVFWR, FPFCWLPTY, MPAFQGWAF, KICHVVFFR, RTYHMIWNR, RVFFWKVQR, MTFVFEARR, KMWQRTFTR, RLWRWRCMR, RINFCFYVR, RQRAILMYR, KMRVWRHQR, RMNLITWHR, FHDQTIFCL, MHDHYCFVL, IPMRRRRSL, FHFCITFCL	See Table 3	See Table 3	P
**CD4+** **T CELL EPITOPES VACCINE COMPONENT**			
DDELFHYFLWTFFFIDLLYAVM	Hypothetical protein	XP_816368.1	P
YIFIECFQIMRAFRLRGASFF	Co-chaperone GrpE	XP_804093.1	P
**B CELL EPITOPES VACCINE COMPONENT**			
GTDEGLLLPVDNDGDESS	Lipophosphoglycan biosynthetic protein/HSP90 superfamily	XP_818651.1	E
QNKKFFADKPDESTL	Kinetoplastid membrane protein 11 (KMP11)	XP_810488.1	P
EGESRHRTRSGSARHHRRHHRNEAGG	Hypothetical protein, conserved	XP_814578.1	P
RRRRHSRSKRGEEDGGET	Hypothetical protein, conserved	XP_814578.1	P
GTPSRTTGRSTSTTRGVSRPTNGVTPSTSLAHRASTPGRTGTRSTTPSRSSVLS	Associated kinase of Tb14-3-3, putative	XP_819464.1	P
GVCTSAEPRDLLDPVALCMPYPGAERIIG	Associated kinase of Tb14-3-3, putative	XP_819464.1	P
NSQETPDQQKTGITRV	Associated kinase of Tb14-3-3, putative	XP_819464.1	P
REARGTSTPRRAETPSGGSRVRGA	Associated kinase of Tb14-3-3, putative	XP_819464.1	P
KRSRSHNDGPARKRRRKDNRP	Methyl-transferase, putative	XP_803963	P
NPSASPEASWQLNQSWNPL	Hypothetical protein, conserved	XP_809003.1	P

a *Antigen name and ID reference obtained at NCBI database upon BLASTP analysis of the corresponding ORF_AA sequence as detailed in Materials and Methods*.

b *Src, source of the epitopes: P, de novo predicted; E, experimentally validated retrieved from IEDB*.

**Figure 3:**
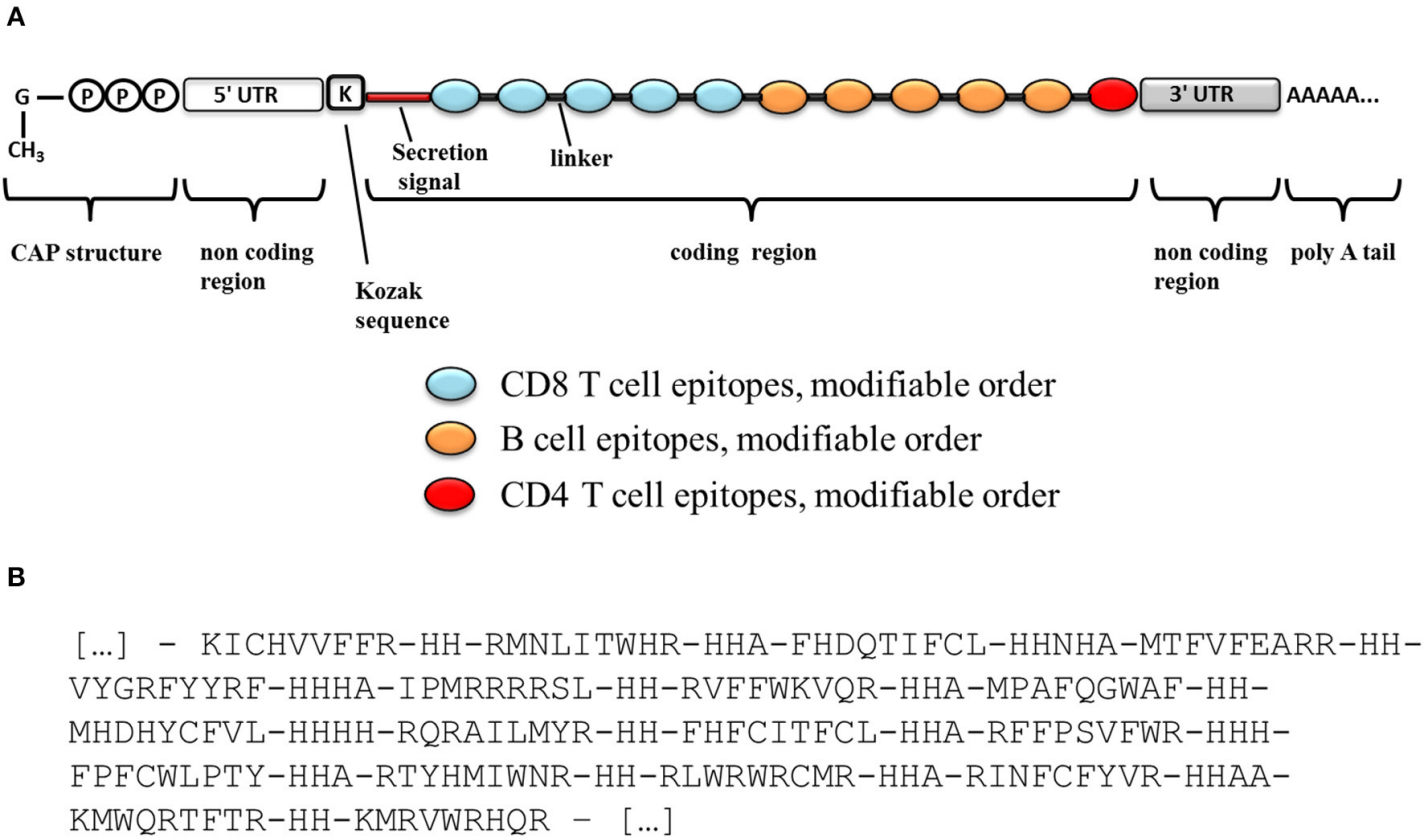
Schematic diagram of an mRNA vaccine construct. **(A)** Schematic diagram of a candidate mRNA construct. **(B)** Optimal organization and ordering of CD8^+^ T cell epitopes part of the vaccine, as well as optimal linker sequences as determined with OptiVac tool (68).

Such epitopes lists could be delivered as independent or concatenated peptides (65), or as genetic constructs in the form of “string-of-beads” (66). However, peptide-based immunization has been deemed with poor immunogenicity indexes (65). Therefore, a genetic immunization seems more appealing, either in the form of plasmid DNA (pDNA) or messenger RNA (mRNA). In this choice, pDNA-based delivery still faces safety concerns, whereas recent advances in the synthesis, manipulation, and immunization with mRNA have made of it a very promising technology that can yield superior immunogenicity than pDNA without the safety issues associated to this (67). However, unlike pDNA, which is transcribed inside a cell into a fully functional mRNA from only a coding sequence, an mRNA has to be carefully designed to contain all necessary elements. A schematic diagram of a potential mRNA-based candidate construct is shown in Figure 3A. The mRNA is usually obtained by *in vitro* transcription from a linearized plasmid, which encodes all the structural elements of a functional mRNA. *In vitro* mRNA structure has been described in detail in the literature (67). Particularly, efficient translation requires a functional 5′-cap structure that must be enzymatically added after the transcription step. The natural 5′-cap is a 7-methyl-guanosine linked to the mRNA by a 5′-5′ tri-phosphate bond, but synthetic analogs called ARCAs (anti-reverse cap analogs) have proven to result in superior translational efficiency and are often currently used. As stability is a major concern with mRNA molecules, several features may increase the stability and/or translation rate of mRNA. Particularly, the incorporation of 5′- and 3′-UTRs (untranslated regions) like those of β-globin will help to increase the translation rate and stability of the *in vitro* transcribed mRNA. In addition, the incorporation of a Kozak sequence, which plays a major role in the initiation of the translation, will also contribute to the mRNA stability and translation efficiency. Finally, the *in vitro* transcribed mRNA must contain a long polyadenosine monophosphate tail (polyA; ideally 120–150 nucleotides long) that further regulates its stability and translational efficiency. This polyA tail can be either included in the template plasmid before transcription or enzymatically added using recombinant poly(A)-polymerase after transcription.

Besides, if epitopes are to be delivered as a genetic “string-of-beads,” linker sequences will be required to space them, precluding the formation of neo-epitopes that would distract the desired immune response (69, 70). We used the tool implemented by Schubert and Kohlbacher to optimally pre-determine the sequence and length of those spacers for HLA I-restricted epitopes (68) (Figure 3B). Taking into consideration the relevance of a prominent CD8^+^ T cell response against the parasite, we would place these epitopes first in the string as those sequences located closer to the 5′-end of the construct would be expressed at higher levels, thus inducing better immunogenic responses (71) (Figure 3A).

**Figure 4:**
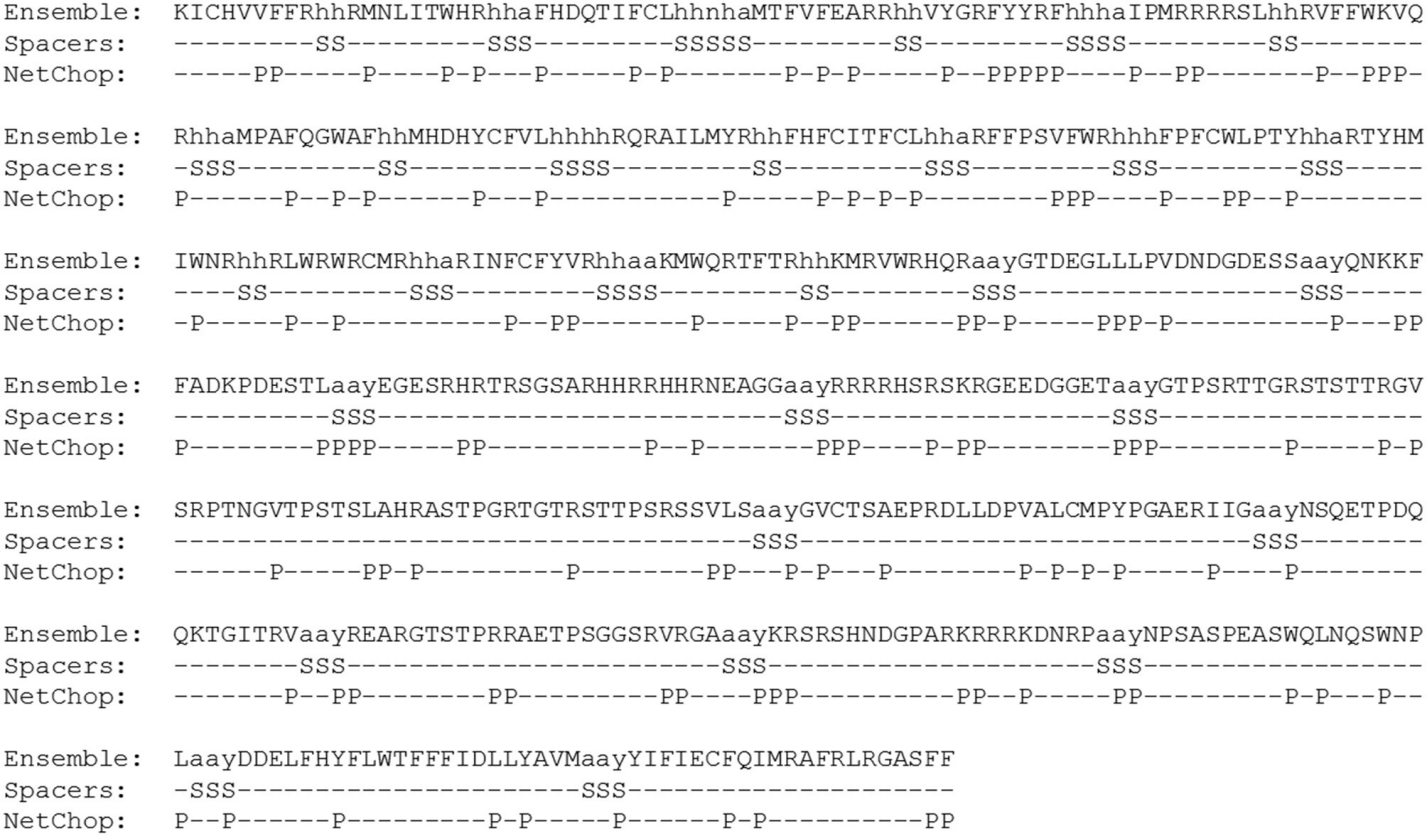
NetChop predictions of proteasome processing in the full peptide sequence of the vaccine ensemble. S, spacers; P, NetChop predictions.

**Figure 5:**
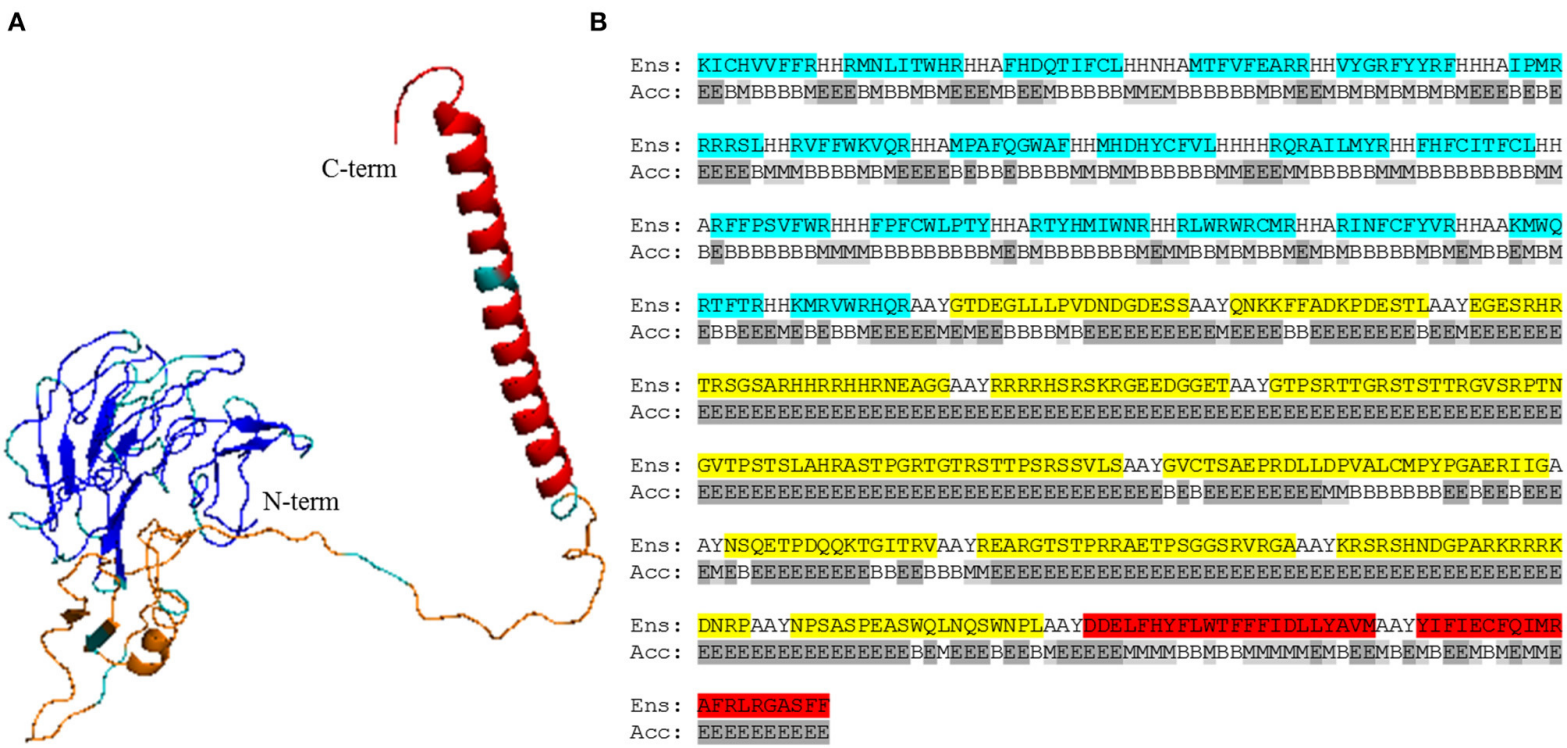
RaptorX modeling and accessibility calculations of the epitope-containing ensemble. **(A)** 3D model of the ensemble, respectively, encompassing from N-term to C-term the listed CD8^+^ T cell epitopes (blue), B cell epitopes^*^ (orange), and CD4^+^ T cell epitopes (red). Linkers are shown in gray. ^*^, B cell epitopes QNKKFFADKPDESTL, EGESRHRTRSGSARHHRRHHRNEAGG, RRRRHSRSKRGEEDGGET, GTPSRTTGRSTSTTRGVSRPTNGVTPSTSLAHRASTPGRTGTRSTTPSRSSVLS, and KRSRSHNDGPARKRRRKDNRP were not modeled by RaptorX as they were considered disordered. **(B)** Full peptide sequence of the vaccine ensemble (Ens) aligned with RaptorX-provided solvent accessibility (Acc) per position. CD8^+^ T cell epitopes are shaded light blue; B cell epitopes are highlighted in yellow; and CD4^+^ T cell epitopes are in red. B, buried; M, medium (light gray); E, exposed (gray).

We selected B cell epitopes paying attention to their subcellular localization in the surface of the parasite and thus they should be preceded by a surface secretory signal. Whether this signal has to be placed at the 5′-end of all the epitopes or just before the B cell epitopes chain will have to be determined. Regarding CD4^+^ T cell epitopes, they will be functional as far as they are physically linked to the others, so they could be placed toward the 3′-end of the construct linked in “the string-of-beads” (Figure 3A). The position, order, and spacing of the B cell and CD4^+^ T cell epitopes will require a detailed experimental evaluation. In an attempt to anticipate this task, we included AAY linkers between B cell and CD4^+^ T cell epitopes (69) and submitted the ensemble sequence to NetChop proteasome processing predictor (56), as well as to VaxiJen v2 and RaptorX web portals to, respectively, obtain its antigenicity score and 3D modeled structure (57, 58). NetChop predicted that proteasome processing is shown in Figure 4. The calculated antigenicity score of the ensemble was 0.68, indicating that the construct was a probable antigen. In the retrieved 3D model, the unstructured organization of the suggested spacers can be observed, whereas the majority of the CD8^+^ T cell epitopes adopt a β-sheet secondary conformation (Figure 5A). In contrast, the two CD4^+^ T cell epitopes lead to the formation of an α-helix, which localizes to the C-term (Figure 5A). With respect to the B cell epitopes, the majority of those that could be modeled did not adopt a defined secondary structure (Figure 5A). The 3D configuration of half of them was not modeled by RaptorX as it reported them as disordered. These were QNKKFFADKPDESTL, EGESRHRTRSGSARHHRRHHRNEAGG, RRRRHSRSKRGEEDGGET, GTPSRTTGRSTSTTRGVSRPTNGVTPSTSLAHRASTPGRTGTRSTTPSRSSVLS, and KRSRSHNDGPARKRRRKDNRP. RaptorX also reported the solvent accessibility (Acc) classification of each position in the ensemble (Figure 5B). Remarkably, such Acc was described as maximum (exposed, E) for 86.7% of the residues within B cell epitopes, which would imply that they are accessible to B cell receptor/antibody recognition (Figure 5B). It was medium-exposed (M) to E in the case of CD4^+^ T cell epitopes (83.7%), and predominantly a buried (B) Acc was calculated for CD8^+^ T cell nonamers (63.0%) (Figure 5B).

## Discussion

Similarly to what has been suggested for leishmaniasis vaccines (72), a vaccine for Chagas disease should fulfill the following requirements: (i) it must be safe; (ii) it will have to induce long-term protection against the wide diversity of *T. cruzi* strains; (iii) such protection will need to be population broad and it should be achieved with a minimum of immunizations; (iv) it will have to be developed and produced very cost-effectively; and ideally (v) it should be effective prophylactically and therapeutically.

The computational-assisted modular design of epitope-based vaccines permits approaching some of those requirements from the very start of the developmental process. In this manuscript, we have applied several *in silico* methodologies to the identification of epitopes of *T. cruzi* toward the design of an epitope-based vaccine for Chagas disease (Figure 2). Nonetheless, such strategy could likewise be explored for the identification of epitope sequences of interest in other complex pathogens responsible for chronic pathologies difficult to intervene against, like *Leishmania* spp. In the case of chronic persistent infections, regarding safety compliance, cross-reactivity between the pathogen and the host is a feature that can be explored in advance computationally. Such cross-reactivity between *T. cruzi* and human antigens has been described and for many years fueled the erroneous assumption that autoimmunity was the sole cause of Chagas disease pathogenesis (73). Thus, the epitope sequences we have prioritized into the ensemble are <70% identity to any protein present in humans, including those of the human microbiome. Nonetheless, we should be cautious with respect to the occurrence of cross-reactivity. Currently available data indicate that occasionally a single mutation can disrupt the antigen recognition process (74), whereas it is possible that cross-recognition may occur even with low similarity levels (75). The structural mechanisms of epitope recognition, which could largely contribute to better understand these features, are yet under debate. Overall, it is common to think that the lower the identity, the better it will be toward avoiding any potential cross-reactivity. However, there is not a study specifically addressing what percentage of similarity removes the chance for such cross-reactivity in the context of epitopes vs. self-peptide recognition (76). Notwithstanding, if we look at the epitope cross-reactivity phenomenon as a matter of ligand–receptor complementarity, this is comparable to the self-complementarity process within folded proteins. There, a 70% threshold value is used for protein sequence analysis (77). Such cutoff has been described as conservative by an approach aimed at reducing epitope redundancy, particularly in relation to long epitopes such as CD4^+^ T cell and B cell epitopes (78). Regarding nonameric CD8^+^ T cell epitopes, it has been described that residue positions 2 and 9 are key to determine epitope binding to MHC-I, whereas residues 3–6 and 8 are engaged in T cell receptor (TCR) motif recognition (79). Thereby, we think that establishing a <70% cut-off, we are restricting the selection of any *T. cruzi* epitopes that are identical to peptides present in humans in more than six (out of nine) residues. Then, at least one of those key positions will always be different in any of the CD8^+^ T cell epitopes progressed by us to any peptide sequence of that length present in humans. Nonetheless, as it is mentioned above, cross-recognition may still occur with low similarity, so any potential cross-reactivity will have to be carefully addressed. We expect that this could be better anticipated by computational tools in the near future as structural comprehension of the epitope recognition process advances. The rationale of applying this criterion of selecting non-identical sequences to proteins in the host susceptible to vaccination has another advantage, as it lines up with the hypothesis that any peptide sequence that is non-conserved in the host will likely be more immunogenic, which is certainly a desirable attribute of any vaccine. Indeed, a lower ability to induce an immune response has been described for peptides from commensal bacteria of the human microbiome (80).

In the aspiration of inducing protection against *T. cruzi* diverse strains, we did select epitopes that besides being <70% identical to human proteins were pan-conserved to a certain threshold among the parasite strains available at TriTrypDB release 35. The idea is that a single vaccine would cover the parasite's ample phylogenetic and geographic space. The importance of selecting invariant protein regions also brings the concept that evolutionary conserved sequences likely represent biologically relevant moieties of the parasite genome and are therefore good targets for therapeutic intervention as it has been described in viral, bacterial, and other parasitic diseases (25–28, 81). Besides, we prioritized T cell epitopes accordingly to their calculated PPC in the target population, i.e., Latin American population, so as to predict a maximum coverage. Therefore, thanks to the use of computational tools and immunoinformatics, we can fence in the parasite's genetic diversity and anticipate to some extent the vaccine coverage from a standpoint as early as the selection of the antigens/epitopes that will go in it.

Another key feature, especially in the development of therapeutics for NTDs, is certainly cost-effectiveness. In fact, considering an average Chagas disease prevalence of ~1% in Latin America (82), keeping developmental costs as low as possible will be fundamental (83). In this respect, a computer-guided selection of the antigens/epitopes can definitely aid to save costs as it narrows down the number of them that will have to be evaluated experimentally. In addition, in an epitope-based strategy with multiple epitopes from multiple antigens, epitopes will have to be delivered either as peptides or genetic constructs, which are cheaper to produce than recombinant proteins or vectors. Nevertheless, vaccine efficacy will be fundamental to determine its final cost-effectiveness (83), and this is something that remains to be determined experimentally.

Vaccine efficacy is connected to the level of immunogenicity and the induction of long-term (memory) protection. Thus, thinking about the preferred delivery system, an RNA-based strategy might be the most suitable. It would ensure direct cytoplasm processing of the epitopes supporting higher expression efficiencies and stronger immune responses in comparison to protein subunit immunizations or pDNA usage (67). In fact, mRNA vaccine candidates against several infectious diseases like flu, rabies, HIV-1, or Zika virus infection have worked very well in preclinical models and reached clinical testing (67). They all rely on the delivery of complete viral antigens, whereas a parasitic vaccine will require the delivery of multiple antigens that would face size constrains. Here, an epitope-based vaccine encompassing a fine selection of the most desirable peptides can overcome this phenomenon. The RNA-based delivery of epitopes has been clinically evaluated already in search of novel immunotherapies for advanced melanoma patients (84). Although it may be a limitation, in principle, no adjuvant should be inoculated with the vaccine as the RNA backbone is known to have self-adjuvant properties (67). Although not including an adjuvant would be desirable for a Chagas disease vaccine as it would contribute to save costs, whether an adjuvant will be required or not will need to be determined experimentally.

Despite the fact that the computer-guided design of vaccines offers a series of advantages, a caveat of our approach is that only linear peptide epitopes can be computed, which excludes conformational and glycan epitopes from the ensemble. The outcome of the two-sided strategy that consisted in crossing experimentally described unique *T. cruzi* epitopes (from IEDB) with the invariant parasite proteome (“IEDB × INVARIANT PROTEOME”) barely yielded a single B cell epitope (Table 2). We had hoped to recover more sequences, but this result was not surprising at all given the limited number of *T. cruzi* antigens studied. Unique *T. cruzi* T cell epitopes from positive assays at IEDB originated from 45 distinct antigens (Tables S3A,B), whereas the 2000 unique B cell epitopes retrieved originated from a total of 133 distinct antigens (Table S3C), meaning that the majority of the parasite protein space is yet unexplored. As a result, we were obliged to a *de novo* prediction of epitopes. In truth, we applied highly restrictive thresholds and only used the very top ranked predicted sequences, but the first thing to do next will be to validate them experimentally.

The antigens of origin of over half of the epitopes selected appeared annotated as hypothetical proteins, which further conveys the need to increase the knowledge about the parasite protein space. These proteins will have to be further studied and annotated, but the fact that their sequences are highly conserved among *T. cruzi* genomes seems to suggest that they are meant to play an important role in the parasite's biology. Among those antigens that had functional annotation, it is worth to highlight KMP11, which has been previously described to be immunogenic and protective (63, 85). The rest were mostly putative enzymes with likely intracellular sub-localization, a feature not surprising given the conservancy criteria used to select the epitopes. In addition, the majority of the selected epitopes were from proteins that had evidences of expression specific to the mammalian infective stages of the parasite (see Tables 2–5). Nonetheless, we found that a few of them were described to be slightly higher expressed [by transcriptomic microarray with the Brazil strain; (62)] in epimastigotes than in the rest of the stages (see Tables 3–5). Since one of such antigens was KMP11 (Table 5), which has been thoroughly studied and known to be the origin of several immunogenic epitopes, we decided to keep those epitopes in.

In comparison to other genome screening of *T. cruzi* epitopes for vaccine design (86), the epitopes included in this ensemble are human epitopes or predicted in the human immunological context. That is, they either were predicted to bind to human MHC class I or class II molecules or have been validated with clinical samples in the case their origin is at IEDB (only B cell epitope GTDEGLLLPVDNDGDESS). This will likely be advantageous for a rapid progression of the candidate, but it entails that at the preclinical developmental stage, the ensemble will require to be tested in humanized mice, for instance, the transgenic strains already available (87–89).

In an epitope-based vaccination strategy with multiple epitopes from multiple antigens, each of those epitopes represents an independent immunological entity. In the case of a Chagas disease vaccine, the ensemble must include CD8^+^ and CD4^+^ T cell epitopes as well as B cell epitopes (8–11), and thus their positioning and ordering within the construct will have to be carefully studied. We have used the tool developed by Schubert and Kohlbacher for the design of the “string-of-beads” fraction with CD8^+^ T cells epitopes (68, 70), which take into account not only the optimum order of the epitopes, but also the type and length of the linker sequences separating each pair of them. The relevance of adequately choosing the spacers resides in that they can be immunogenic themselves or give rise to novel epitopes which would preclude or downsize the desired immune response. Following the work by Velders et al. (69), we have included AAY linkers between B and CD4^+^ T cell epitopes, and they were adequately predicted by NetChop in the ensemble proposed (Figure 4). Nonetheless, epitopes' ultimate ordering and localization as well as that of any spacers in between will have to be empirically determined. Similarly, taking into account that the immunogenicity of isolated epitopes could change when they are put together in a string of beads array, although VaxiJen v2 score for both constructs was above the threshold, their overall immunogenicity will have to be carefully studied experimentally.

Ideally, the availability of a Chagas disease vaccine would substitute BNZ and NFX treatments. However, the co-administration of vaccine and drug is also being investigated as the former could contribute to reduce the drug's dosage, and thus its related toxicity, while maintaining its efficacy as described recently (90). In any case, vaccination is arguably the most successful biomedical intervention ever developed against infectious diseases (91), and reverse vaccinology was shown to be fundamental toward the successful development of a multi-antigen vaccine against the otherwise difficult-to-tackle pathogen meningococcus serogroup B (92). Multi-antigen vaccines might also be the answer to obtain immunotherapeutic products against other biologically complex pathogens such as the parasites responsible for many of the currently recognized NTDs, whose treatment still relies on toxic chemotherapies (93). The advent of next-generation sequencing has allowed access to genomics information of several of these pathogens, like *T. cruzi*. For many of them, the availability of vaccines would mean a major breakthrough in their clinical management and therefore stronger efforts made in this direction would surely pay back.

## Conclusions and Limitations

Chagas disease, a neglected infectious disease caused by the parasite *T. cruzi*, exerts a huge burden in Latin America. In recent decades, its impact has been globalized mainly to North American countries and Europe. Upon a generally asymptomatic acute stage, the infection becomes persistent and it is in its chronic stage when the disruption of heart and/or gut tissues occurs. The damage to these tissues can be fatal if untreated and up to 30% of those chronically infected are estimated to end up developing these symptoms. Despite BNZ and NFX being available to treat the infection, their efficacy is reduced at the chronic stage and both have frequent adverse effects associated. In this context, the availability of a vaccine that could prevent the infection or the development of the symptomatology would be a major breakthrough for the clinical control of the disease. Several efforts have been initiated in this respect, but the biological complexity of the parasite and the lack of funding to progress any candidate beyond preclinical testing have hindered the matter. We herein propose an alternative vaccine approach based on epitopes from a range of different parasite antigens to cope with the parasite's antigenic complexity. The epitopes were prioritized considering their conservation among the distinct *T. cruzi* sequenced genomes so as to provide a single pan-vaccine, which would contribute to save developmental costs. Besides, the proposed epitope-based vaccine candidate would be delivered in the form of a genetic construct that can be synthetized at a fraction of the cost of producing recombinant protein subunits or virus vectored antigens. The provided ensemble would elicit both cellular and humoral immunity, required to control the intracellular and extracellular forms of the parasite (amastigotes and trypomastigotes, respectively) in the mammalian host. The T cell component consists of 18 CD8^+^ T cell epitopes from 17 distinct antigens and 2 CD4^+^ T cell epitopes from another 2 antigens that could elicit a cellular response in virtually the whole target population of such a vaccine. On the other hand, the B cell component of the vaccine encompasses a total of 10 epitopes from six different surface-exposed antigens that could elicit antibodies against the free swimming trypomastigote stage. Notably, all the epitopes in the ensemble were prioritized in agreement with their lack of identity to any protein sequences present in humans in order to preclude cross-reactivity reactions thus favoring the safety of the vaccine candidate.

We initially attempted to identify conserved epitopes that had been experimentally validated and deposited in the IEDB database, but it happened that only one of the *T. cruzi-*specific unique epitopes at IEDB (B cell epitope GTDEGLLLPVDNDGDESS) could be rescued out of the conserved *T. cruzi* proteome generated. Therefore, we had to perform *de novo* predictions of epitopes departing from that conserved proteome. Aware of the limitations of this procedure, we were very stringent and only selected top qualifying predicted epitopes. Nonetheless, whether they are processed and presented in the course of an infection by *T. cruzi* remains to be demonstrated. Even if immunogenic, we would still not know to what extent the here presented vaccine ensemble could provide protection from *T. cruzi* infection. This is something that we will have to evaluate in forthcoming experiments upon validation of the epitopes.

In relation to the T cell component, we assumed population coverage estimates based on peptide binding predictions to MHC molecules. Although the reliability of peptide–MHC binding predictions has been widely proved, this is a feature that will need further testing. An appropriate processing of the antigens is a key feature toward the immunogenicity of the epitopes, and this will have to be thoroughly studied in relation to the delivery of the epitopes in the form of a genetic construct. We have used a computational tool to provide the optimum ordering and spacing of the CD8^+^ T cell epitope component in the suggested construct. Nonetheless, this will require further proof as well as it will be necessary to evaluate which is the optimum order and spacing to achieve maximum immunogenicity with the CD4^+^ T cell and B cell epitopes. In relation to the latter, we do not provide any glycan-based epitopes as they cannot be extracted with the protein-based reverse vaccinology procedure followed. We neither include any conformational epitopes since these cannot be isolated from their context and we only focused on linear epitopes. It needs to be tested whether these epitopes elicit antibodies that are capable of recognizing the native protein conformations. Omics- data repositories such as EuPathDB and IEDB (94, 95), along with the series of immunoinformatics tools described in this work, are extremely useful to perform *in silico* studies that can help to guide wet lab experiments contributing to save time and money. Nonetheless, the next step will be to take these epitopes onto *in vitro* immunological assays to validate them and determine their immunogenicity and then devise challenge-protection preclinical studies to ultimately qualify the strategy.

## Data Availability Statement

The data used to support the findings of this study are included within the article or within the supplementary information file(s). The scripts developed are available from the corresponding authors upon request.

## Author Contributions

LM-T, PR, and JA-P conceived the study. LM-T and PR wrote the scripts. LM-T and JA-P analyzed the data and wrote the article. PB provided Figure 3. M-JP and JG supported the research. All authors revised the manuscript and edited its final version.

### Conflict of Interest

The authors declare that the research was conducted in the absence of any commercial or financial relationships that could be construed as a potential conflict of interest.

## Supplementary Material

Supplementary File 1 List of MHC Class I alleles that were used for the prediction of binding to MHC I and the calculation of the PPC provided by CD8^+^ T cell epitopes.

Supplementary File 2 Multiple sequence alignment (MSA) of *T. cruzi* KMP11 protein sequences of the eight parasite strains included in the study and the structure-based KMP11 predicted epitope. We used MUSCLE (v3.8) to get the MSA (34).

Additional Table 1 This Excel file includes Tables S1A–C, which, respectively, contain *T. cruzi*-specific and unique CD8^+^ T cell epitopes, CD4^+^ T cell epitopes, and B cell epitopes elicited in the course of a natural infection as reported in IEDB.

Additional Table 2 Table S2 contains those IEDB unique *T. cruzi-*specific B cell epitopes elicited in the course of a natural infection that are also conserved (*H* > 1.0), as described in section Identification of *T. cruzi* Conserved Experimentally Validated Epitopes at IEDB.

Additional Table 3 This Excel file includes Tables S3A–C, which, respectively, contain all the predicted *T. cruzi*-specific and conserved CD8^+^ T cell epitopes, CD4^+^ T cell epitopes, and B cell epitopes as described in sections Prediction of T Cell Epitopes and Prediction of B Cell Epitopes.
