# Corrigendum: *In silico* Design of an Epitope-Based Vaccine Ensemble for Chagas Disease

**DOI:** 10.3389/fimmu.2019.03124

**Published:** 2020-01-29

**Authors:** Lucas Michel-Todó, Pedro Antonio Reche, Pascal Bigey, Maria-Jesus Pinazo, Joaquim Gascón, Julio Alonso-Padilla

**Affiliations:** ^1^Barcelona Institute for Global Health (ISGlobal), Hospital Clínic, University of Barcelona, Barcelona, Spain; ^2^Laboratory of Immunomedicine, Faculty of Medicine, University Complutense of Madrid, Madrid, Spain; ^3^Université de Paris, UTCBS, CNRS, INSERM, Paris, France; ^4^PSL University, ChimieParisTech, Paris, France

**Keywords:** Chagas disease, *Trypanosoma cruzi*, epitope-based, vaccine, CD4 T cell, CD8 T cell, B cell epitopes

In the original article, the reference for “77” was incorrectly written as “Batu S, Bhattacharyya D, Banerjee R. Self-complementarity within proteins: bridging the gap between binding and folding. *Biophys J*. (2012) 102:2605–14. doi: 10.1016/j.bpj.2012.04.029.” It should be “Basu S, Bhattacharyya D, Banerjee R. Self-complementarity within proteins: bridging the gap between binding and folding. *Biophys J*. (2012) 102:2605–14. doi: 10.1016/j.bpj.2012.04.029.”

The authors apologize for this error and state that this does not change the scientific conclusions of the article in any way. The original article has been updated.

